# Copeptin as a dual biomarker in type 2 diabetes: association with glycemic control and diabetic kidney disease

**DOI:** 10.1186/s12902-026-02182-5

**Published:** 2026-02-19

**Authors:** Nehal Kamal Rakha, Ahmed Fayed, Shady A. Ramiz, Eman ElSayed, Sameh A. Al-Dawy, Fadia Moris Bolis, Ahmed Fathy, Sameh Abouzeid, Rasha Ahmed Darwish, Mohammed Gomaa, Hend A. Elsheimy

**Affiliations:** 1 Nephrology Unit, Internal Medicine Department, Kasr Alainy School of Medicine, Cairo University, Cairo, Egypt; 2 Nephrology Department, Theodor Bilharz Research Institute, Cairo, Egypt; 3 Endocrinology Unit, Internal Medicine Department, Kasr Alainy School of Medicine, Cairo University, Cairo, Egypt; 4 Cairo University Hospitals, Al-Saray St., El-Maniel, Cairo, 11562 Egypt

**Keywords:** Copeptin, Type 2 diabetes, Glycemic control, Diabetic kidney disease

## Abstract

**Background:**

Chronic kidney disease and end-stage renal disease are significantly contributed by diabetes mellitus (DM), especially diabetes mellitus type 2 (T2DM). A stable surrogate marker of vasopressin, the serum copeptin, has become a potential biomarker of poor glycemic control and diabetic kidney disease (DKD). The research objective is to estimate the usefulness of the serum copeptin as a marker of glycemic status and its correlation with DKD in patients with T2DM.

**Methods:**

This cross-sectional study was conducted on 60 participants at Cairo University Hospital between 2021 and 2022. Participants were divided into 40 T2DM patients (26 males, 14 females; mean age 52.62 ± 9.7 years) and 20 healthy controls. Diabetic patients were further categorized into controlled (HbA1c ≤ 7%, *n* = 18) and uncontrolled (HbA1c > 7%, *n* = 22) subgroups. Clinical evaluation, biochemical tests, and serum copeptin levels (measured via ELISA) were analyzed. Cairo university, Faculty of Medicine, Ethical approval: N-247-2023.

**Results:**

Serum copeptin levels were significantly higher in diabetic patients compared to controls (*p* < 0.001), and in uncontrolled vs. controlled diabetics (*p* < 0.001). Copeptin positively correlated with fasting glucose, HbA1c, serum creatinine, and urinary protein, and negatively with eGFR. ROC analysis identified copeptin as a sensitive marker for both poor glycemic control at a cut off value > 3452 pg/mL (AUC 0.925) and DKD at a cut off vlue > 370 pg/mL (AUC 0.94), both with high sensitivity and specificity.

**Conclusion:**

The level of serum copeptin correlates positively with poor glycemic status as well as DKD among T2DM patients. It can be an effective, non-invasive biomarker used in early diagnosis and risk stratification of diabetic complications.

**Clinical trial registration:**

Not applicable.

## Introduction

Diabetes mellitus, particularly type 2 diabetes mellitus (T2DM), is one of the most important public health challenges worldwide; in 2019 an estimated 463 million adults suffered from diabetes and by 2045 this number is expected to increase to 700 million. This increasing burden is associated with significant health, social and economic consequences and estimates suggest that low- and middle-income countries, such as those in the Middle East and North Africa region (MENA), will see some of the most marked increases in prevalence [1].

Diabetic kidney disease (DKD) affects approximately one-third of those with diabetes and is the most common cause of ESRD in the world, accounting significantly for cardiovascular related morbidity, mortality and cost to healthcare. The pathogenesis of DKD is characterized by multifaceted interplay among several components such as chronic hyperglycemia, glomerular hyperfiltration and intraglomerular hypertension, oxidative stress, activation of the renin–angiotensin–aldosterone system, pro-fibrotic and pro-inflammatory signaling pathways. Conventional biomarkers of injury, such as albuminuria and estimated glomerular filtration rate (eGFR), largely capture the presence of advanced structural injury to the kidney, precluding risk stratification at early stages or intervention prior to extensive loss of function [2].

A mechanically representable axis between fluid balance, hemodynamics and metabolic and renal outcomes is the vasopressin system. Copeptin, the C-terminal part of pre-pro-vasopressin, is released in equimolar concentrations with arginine vasopressin (AVP) and represents a stable and reliable surrogate marker for circulating levels of AVP. High copeptin has been linked with incident chronic kidney disease (CKD), accelerated eGFR decline, progression to end-stage renal disease (ESRD) in those with diabetes and/or DKD populations [3].

Chronic low-grade inflammation is an important driver of insulin resistance, endothelial dysfunction, and the progression of DKD. Several inflammatory markers (e.g., C-reactive protein, leukocyte-based ratios, platelet indices, uric acid-related markers) are associated with adverse metabolic and renal trajectories. Experimental and clinical data indicate that the vasopressin/copeptin axis is responsive to systemic stress and inflammatory stimuli; therefore, copeptin may be positioned as an integrative marker at the intersection of inflammation, metabolic dysregulation, and renal injury. In addition to this fact in both diabetes and CKD cohorts, higher levels of copeptin predict major adverse cardiovascular events arterial stiffness and subclinical atherosclerosis which supports its role as a cardiovascular risk marker for such a high-risk population [4, 5].

In patients with diabetes, elevated copeptin levels have been associated with poor glycemic control, insulin resistance, albuminuria, and rapid deterioration of kidney function. Copeptin may independently predict the development of DKD and cardiovascular events. Observational cohorts in T2DM patients with albuminuria indicate that copeptin enhances risk stratification for cardiovascular outcomes beyond traditional risk factors; hence there is potential value for its integration into multimarker panels in DKD. However, despite increasing evidence to support this fact, relatively few studies have assessed copeptin jointly concerning glycemic control and severity of DKD within the same T2DM cohort - particularly from high burden regions like Egypt where prevalence rates of diabetes as well as DKD-related ESRD are rising rapidly [6, 7].

This makes it clinically relevant to describe in detail how copeptin behaves as a biomarker that reflects both metabolic dysregulation and early renal injury in T2DM. If the data show this then it will be clear if copeptin can improve risk stratification for poor glycemic control and DKD beyond what conventional markers offer. Such information would also help identify those individuals who might benefit from intensified therapeutic strategies or closer renal surveillance [8].

This study focuses on measuring serum copeptin levels in adults with T2DM and healthy controls, looking at how it is related to glycemic control and DKD parameters, as well as finding diagnostic cut-off values for predicting poor metabolic control and kidney dysfunction through receiver operating characteristic analysis.

## Materials and methods

### Study design and setting

This observational cross-sectional study was conducted at the Department of internal medicine, Cairo University Hospital, Egypt, during the period from 2021 to 2022. We have enrolled 60 participants and divided them into two major groups with 40 diabetic patients and 20 healthy controls. Post-hoc power analysis: using observed copeptin means and SDs between diabetics and controls (Cohen’s d ≈ 5.28), post-hoc power > 99% at α = 0.05. For conservative planning (d = 0.75), the current unbalanced allocation (40 vs. 20) yields ~ 77% power; to reach 90% power for d = 0.75, ≈ 39 participants per group would be required. The sample size generates acceptable statistical power in the main aims of the study. The very large effect size observed (Cohen’s d ≈ 5.28) should be interpreted with caution as it most likely mirrors the compounded influences of severe metabolic derangement, renal impairment, and intergroup heterogeneity rather than any clean biological signal that could be solely attributed to copeptin.

### Study population

1. *Diabetic Group (n = 40)*
  This group included type 2 diabetic patients with a disease duration of more than 5 years. The mean age was 52.62 ± 9.7 years with male female proportions of 26:14. The diabetic subjects were then classified according to glycemic control employing baseline HbA1c levels into:
  Controlled diabetes mellitus (Group 1 A, *n* = 20; Patients in good glycemic control (HbA1c ≤ 7%).
  Uncontrolled Diabetes (Group 1B, *n* = 20) Patients with poor glycemic control (HbA1c ≥ 7%).
  subgroup analyses by sex and age tertiles were planned but limited by sample size.
2. *Healthy Control Group (n = 20)*
  This group was composed of seemingly healthy people as recruited from the working staff of the Department of Internal Medicine. All the controls were sex and age-matched to the diabetic group and did not have records of diabetes, hypertension, and renal disease.
  Participants, with any of the diseases listed to have effects on serum copeptin, were disqualified: Sepsis or septic shock, acute exacerbation of COPD, diabetes insipidus, autosomal dominant polycystic kidney disease (ADPKD), acute myocardial infarction and Cerebrovascular stroke.

### Methods

All participants underwent a detailed medical history review and physical examination. The following investigations were performed: complete blood count (CBC), fasting blood glucose, post-prandial glucose, HbA1c, serum creatinine, blood urea nitrogen, uric acid, estimated glomerular filtration rate (eGFR), serum and urinary sodium, serum albumin, total protein, and 24-hour urinary protein.

#### Measurement of serum copeptin

The copeptin value of serum was determined via sandwich Enzyme-Linked Immunosorbent Assay (ELISA) kit (Human Vasopressin-Neurophysin 2-Copeptin, EIAab, China; Cat. No. E0462h) [9, 10].

### Statistical analysis

Statistical analysis was performed using SPSS v.20.0. (SPSS Inc., Chicago, IL, USA). Continuous variables were tested for normality using the Shapiro–Wilk test. Normally distributed variables are presented as mean ± SD and compared using independent-samples Student’s t-test; non-normal variables are presented as median (IQR) and compared using the Mann–Whitney U test. Categorical variables were compared with χ² tests. Correlations were assessed using Spearman’s rank correlation coefficient. To account for demographic differences between groups in age, we conducted ANCOVA with copeptin as the dependent variable and group (diabetic vs. control) as the independent factor adjusted for age and sex, and calculated partial correlations adjusted for age and sex. Subgroup analyses by sex and age tertiles were planned but limited by sample size. ROC analyses were performed and AUCs with 95% CI were computed (nonparametric/bootstrap 2000 replicates). For the sensitivity and specificity estimates we report exact (Clopper–Pearson) 95% CIs. Two-sided p-values < 0.05 were considered statistically significant. The statistical significance criterion was fixed as *P* < 0.05 and the highly significant value was *P* < 0.001.

## Results

Significant differences between diabetics and healthy controls are observed in all parameters assessed and depicted in Table 1. Patients with diabetes were older with increased blood pressure, fasting glucose and HbA1c levels which showed poor glycemic control. The patients also had deranged renal functions with an increase in serum urea and creatinine, low eGFR, and higher urinary sodium and protein loss. Most importantly, the level of serum copeptin was highly elevated in diabetics indicating its possible use as a biomarker of poor glycemic control and diabetic kidney involvement (Table 1).

**Table 1:** Demographic and baseline laboratory data of the studied patients

Variables	Diabetic group (*n* = 40)	Control group (*n* = 20)	*P*-value
**Age (**Years**)** (Mean ± SD)	52.62 ± 9.7	30.3 ± 8.8	a 0.000^**^
**Male** (Number (%))	14 (40.6)	9 (53.1)	b 0.000^**^
**Female** (Number (%))	26 (59.4)	11 (46.9)	
**BMI** (kg/m²) (Mean ± SD)	31.17 ± 3.96	29.3 ± 2.05	a 0.107
**Systolic Blood Pressure** (mmHg) (Mean ± SD)	141.1 ± 17.7	114.7 ± 13.3	a 0.000^**^
**Diastolic Blood Pressure** (mmHg) (Mean ± SD)	85.6 ± 11.2	71.0 ± 12.6	a 0.001^*^
**Fasting Glucose (mg/dl)** (Mean ± SD)	119.55 ± 31.36	88.85 ± 4.4	0.000**
**HbA1c (%)** (Mean ± SD)	7.2 ± 1.2	5.3 ± 0.31	0.000**
**Hemoglobin** (gm/dl) (Mean ± SD)	12.37 ± 1.9	11.2 ± 0.92	0.000**
**WBCs** (×10^9^/ml) (Mean ± SD)	7.04 ± 1.5	5.8 ± 1.9	0.006*
**Platelet count** (×10^9^/ml (Mean ± SD)	272.6 ± 73.8	240.75 ± 58.1	0.000**
**Albumin (gm/dl)** (Mean ± SD)	3.71 ± 0.35	3.8 ± 0.36	0.479
**Total Protein** (gm/dl) (Mean ± SD)	6.9 ± 1.2	7.3 ± 0.36	0.137
**Serum Na**^**+**^ (mmol/dl) (Mean ± SD)	133.6 ± 3.9	135.5 ± 6.1	0.256
**Serum Urea** (mg/dL) (Mean ± SD)	64.7 ± 16.13	19.9 ± 9.5	0.000**
**Creatinine** (mg/dL) (Mean ± SD)	2.04 ± 0.83	0.74 ± 0.16	0.000**
**Estimated GFR (mL/min/1.73 m²)** (Mean ± SD)	44.3 ± 19.8	145.7 ± 40.9	0.000**
**24 h Urinary Na**^**+**^ (mmol/L) (Mean ± SD)	36.0 ± 10.8	30.4 ± 9.2	0.02*
**24 h urinary protein** (gm/L) (Mean ± SD)	0.7 ± 0.8	0.04 ± 0.06	0.002**
**Copeptin (pg/ml)** (Mean ± SD)	3746.7 ± 780.3	253.88 ± 294.6	0.000**

BMI= Body mass index; HbA1c= glycated hemoglobin; WBCs = White blood cells; Serum Na^+^= Serum Sodium; GFR = Glomerular Filtration Rate; SD= Standard Deviation

P value < 0.05* significant; P value < 0.001 ** Highly significant; a Independent sample t test

b Chi-square test (X2)

As shown in Table 2, there was consistency in demographic and the duration of diabetes between the controlled and uncontrolled diabetic patients; nevertheless, the uncontrolled diabetic patients had worse metabolic and renal profiles. Their level of fasting glucose, HbA1c was quite high, and they had an increased level of serum urea, creatinine, 24 h urinary proteins and reduced eGFR that suggests higher renal impairment. It is noteworthy that the levels of copeptin were more increased in the uncontrolled group indicating that it could be related to both poor glycemic control and kidney dysfunction. Moreover, in the uncontrolled group, serum sodium levels were lower, whereas urinary output of sodium was increased, fortifying the connection between copeptin, sodium metabolic processes, and illness severity (Table 2).

**Table 2:** Demographic and clinical characteristics of controlled and uncontrolled diabetic groups

Variables	Controlled diabetic Group 1 A (*n* = 20)	Uncontrolled diabetic Group 1B (*n* = 20)	*P*-value
**Age (**Years**)** (Mean ± SD)	52.4 ± 7.6	52.8 ± 11.6	a 0.88
**Male** (Number (%))	12 (60.0)	14 (70.0)	b 0.507
**Female** (Number (%))	8 (40.0)	6 (30.0)	
**Duration of Diabetes (**Years**)** (Mean ± SD)	15.8 ± 8.8	17.8 ± 7.1	a 0.394
**BMI** (kg/m²) (Mean ± SD)	31.5 ± 3.4	30.8 ± 4.4	a 0.557
**Systolic Blood Pressure** (mmHg) (Mean ± SD)	142.1 ± 15.2	140.2 ± 20.22	a 0.789
**Diastolic Blood Pressure** (mmHg) (Mean ± SD)	86.7 ± 8.4	84.5 ± 9.65	a 0.33
**Fasting Glucose (mg/dl)** (Mean ± SD)	95.4 ± 7.2	143.7 ± 027.8	0.000**
**HbA1c (%)** (Mean ± SD)	6.1 ± 0.42	8.3 ± 0.53	0.000**
**Hemoglobin** (gm/dl) (Mean ± SD)	13.4 ± 1.79	12.04 ± 1.90	0.023*
**WBCs** (×10^9^/ml) (Mean ± SD)	7.54 ± 1.6	6.6 ± 1.38	0.097
**Platelet count** (×10^9^/ml (Mean ± SD)	287.9 ± 66.1	257.3 ± 78.1	0.194
**Albumin (gm/dl)** (Mean ± SD)	3.71 ± 0.3	3.7 ± 0.4	0.93
**Total Protein** (gm/dl) (Mean ± SD)	7.27 ± 0.4	6.6 ± 1.2	0.08
**Serum Na**^**+**^ (mmol/dl) (Mean ± SD)	136.0 ± 3.17	133.1 ± 3.77	0.012*
**Serum Urea** (mg/dL) (Mean ± SD)	37.9 ± 10.3	55.6 ± 10.2	0.000**
**Creatinine** (mg/dL) (Mean ± SD)	1.4 ± 0.35	2.6 ± 0.7	0.000**
**Estimated GFR (mL/min/1.73 m²)** (Mean ± SD)	59.59 ± 15.9	29.0 ± 8.0	0.000**
**24 h Urinary Na**^**+**^ (mmol/L) (Mean ± SD)	32.1 ± 9.6	39.9 ± 9.6	0.02*
**24 h urinary protein** (gm/L) (Mean ± SD)	0.6 ± 0.87	1.5 ± 0.89	0.005*
**Copeptin (pg/ml)** (Mean ± SD)	3044.65 ± 288.82	4448.75 ± 358.82	0.000**

BMI= Body mass index; HbA1c= glycated hemoglobin; WBCs = White blood cells; Serum Na^+^= Serum Sodium; GFR = Glomerular Filtration Rate; SD= Standard Deviation

P value < 0.05* significant; P value < 0.001 ** Highly significant; a Independent sample t test

b Chi-square test (X2)

Table 3 indicates that there is a statistically significant relation between an increase of serum copeptin level and both kidney injury and poor glycemic control. 95% of patients with kidney dysfunction had copeptin levels beyond 370 pg/ml, this was not applicable in 5% of healthy controls implying that the copeptin has a close association with kidney dysfunction. Similarly, there was a high prevalence of copeptin > 3452 pg/ml in 90% of diabetics who had poor glycemic control compared to only 5% of controlled diabetics, and this explains how copeptin can be applied as an important indicator of ineffective glycemic control. The results justify the possible establishment of copeptin as a two-fold marker of not only diabetic nephropathy, but also deficient metabolic regulation (Table 3).

**Table 3:** Relation between copeptin level and kidney injury (*n* = 60) and relation between copeptin level in diabetic patients with good and poor glycemic control (*n* = 40)

Variables	Kidney injury (*n* = 40)	Normal control (*n* = 20)	*P* value
**Copeptin > 370 pg/ml (Positive)** (Number (%))	38 (95%)	1 (5%)	0.000
**Copeptin ≤ 370 pg/ml (Negative)** (Number (%))	2 (5%)	19 (95%)	
**Variables**	**Controlled DM (n = 20)**	**Uncontrolled DM (n = 20)**	**P value**
**Copeptin > 3452 pg/ml (Positive)** (Number (%))	1 (5%)	18 (90%)	0.000
**Copeptin ≤ 3452 pg/ml (Negative)** (Number (%))	19 (95%)	2 (10%)	

DM = Diabetes mellitus

Table 4 is worth highlighting remarkable correlations between the serum copeptin and important biochemical indices. Copeptin was found to be highly correlated to HbA1c, fasting glucose, serum urea, creatinine, urinary sodium, and proteinuria (HbA1c (*r* = 0.820, *P* < 0.001), Fasting glucose (*r* = 0.695, *P* < 0.001), Serum urea (*r* = 0.446, *P* = 0.002), Creatinine (*r* = 0.715, *P* < 0.001 To the contrary, it was inversely proportional to serum sodium and eGFR (Serum sodium (*r* = 0.380, *p* = 0.016), eGFR (*r* = 0.707, *p* < 0.001)), hence higher levels of copeptin indicate deteriorating kidney functioning. In general, these associations warrant the role of copeptin as a dual-purpose biomarker of glycemic burden and predisposing renal decline in type 2 diabetes (Table 4).

**Table 4:** Correlation of copeptin with biochemical variables among diabetic patients

Variables	Copeptin level	
	*R*	*P*-Value
**Fasting Glucose (mg/dl)**	0.695	0.000**
**HbA1c (%)**	0.820	0.000*
**Serum Na**^**+**^ (mmol/dl)	-0.380	0.016*
**Serum Urea** (mg/dL)	0.446	0.002*
**Creatinine** (mg/dL)	0.715	0.000**
**Estimated GFR (mL/min/1.73 m²)**	-0.707	0.000*
**24 h Urinary Na**^**+**^ (mmol/L)	0.372	0.04*
**24 h urinary protein** (gm/L)	0.485	0.01**

HbA1c= glycated hemoglobin; Serum Na^+^= Serum Sodium; GFR = Glomerular Filtration Rate

**. Correlation is significant at the 0.01 level (2-tailed)

*. Correlation is significant at the 0.05 level (2-tailed)

**Fig. 1:**
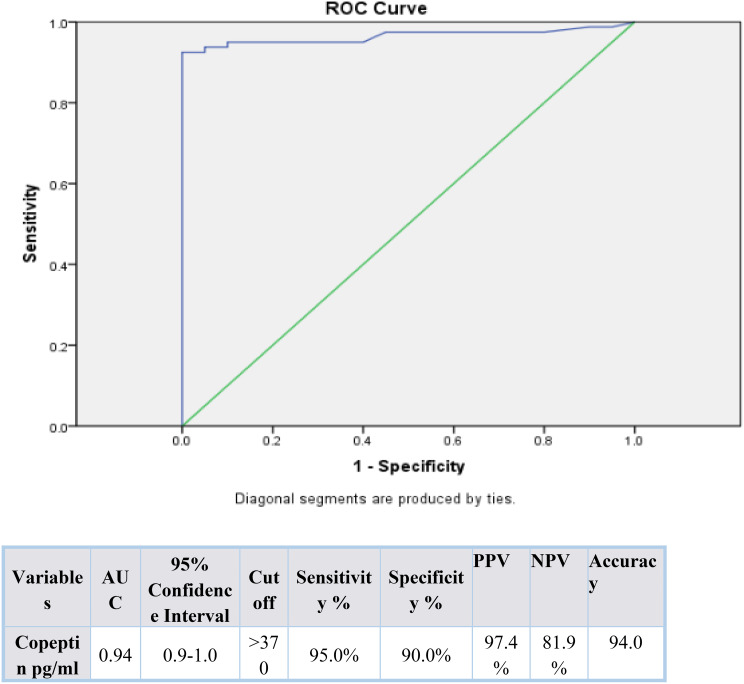
ROC curve for copeptin as a screening marker for kidney injury (*n* = 60). AUC= Area Under the Curve; PPV = Positive predictive value; NPV= Negative predictive value

**Fig. 2:**
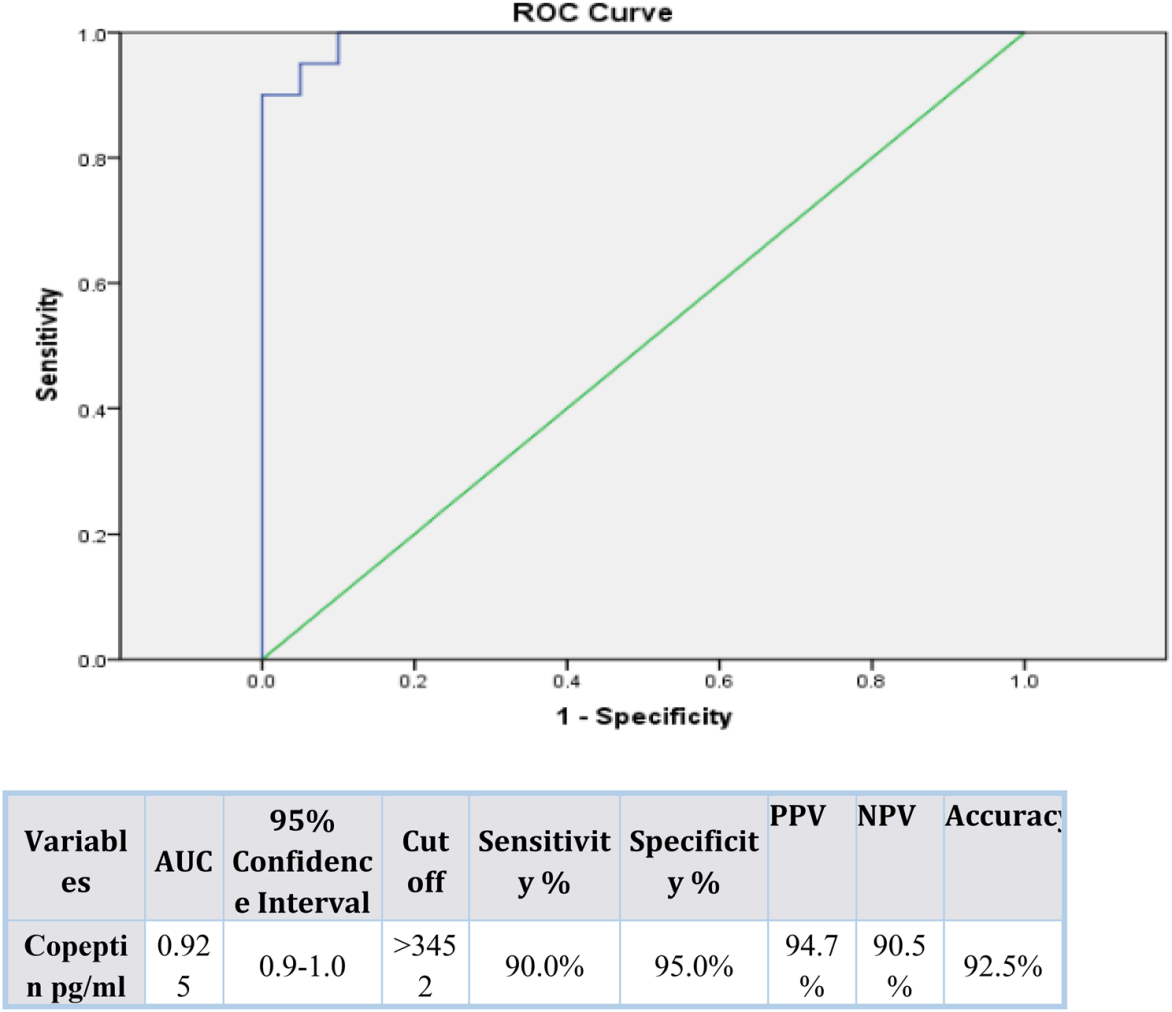
ROC curve for copeptin as a screening marker between diabetic patients with good and poor glycemic control (*n* = 40). AUC= Area Under the Curve; PPV = Positive predictive value; NPV= Negative predictive value

The ROC curve analysis used in Figs. 1 and 2 shows that the serum copeptin is useful in the distinction between patients with good versus those with poor glycemic control among the diabetic cohort, a cut off value > 3452 pg/mL showed AUC 0.925, sensitivity 90%, specificity 95%. Moreover, copeptin cut off value > 370 pg/mL can be used to distinguish patients with diabetic kidney involvement (AUC 0.94, sensitivity 95%, specificity 90%). The high sensitivity and specificity of the above mentioned copeptin cutoff values, proves that copeptin can become a potential marker of poor glycemic control and diabetic kidney involvement. The results also confirm the clinical importance of implementing copeptin measurement in the evaluation and observation of diabetic patients. The marked difference between optimal copeptin cut-off values for diabetic kidney involvement (> 370 pg/mL) and poor glycemic control (> 3452 pg/mL) reflects differences in cohort stratification and outcome definition rather than analytical or assay-related inconsistency.

## Discussion

Copeptin is a glycopeptide derived from provasopressin and is associated with vasopressin and neurophysin II. It serves as a biomarker for vasopressin function and is linked to the regulation of blood glucose and stress responses. Copeptin levels can predict blood sugar changes, indicate baseline blood sugar control, and may be a marker for early glycemic dysregulation. High copeptin levels are associated with decreased pancreatic β-cell function. Stress responses, hyponatremia, sepsis, chronic renal failure, diabetes mellitus, cardiovascular accidents, and acute myocardial infarction can increase copeptin levels [11–14].

In the present study, serum copeptin concentrations were significantly increased in type 2 diabetic patients in comparison to the healthy population, it was also significantly higher in uncontrolled than controlled DM. Copeptin was robustly correlated with indicators of glycemic burden (HbA1c, fasting glucose), renal dysfunction (creatinine, eGFR, proteinuria) and serum sodium.

Our study advances the literature by demonstrating, within a single cohort, that copeptin strongly associates with both poor glycemic control and renal dysfunction and by providing empirically derived ROC cut points for both outcomes (> 3452 pg/mL for poor glycemic control with AUC 0.925, sensitivity 90%, specificity 95%; and > 370 pg/mL for kidney injury with AUC 0.94, sensitivity 95%, specificity 90%). This dual utility complements and extends previous Egyptian and international reports that primarily examined renal outcomes only, offering a translational framework for copeptin as an integrated metabolic-renal risk marker useful in screening for renal injury and poor glycemic control.

In the recent Egyptian data given by El Soudany et al. (2023), the concentration of plasma copeptin in type 2 diabetic patients with nephropathy was greatly increased compared to those without. It also showed a positive correlation with creatinine, urea, albuminuria, and negative relation to eGFR (*P* < 2%) [6]. We have the same findings that support the use of copeptin as a biomarker of diabetic nephropathy.

A larger cohort study (the German CKD Study, 2023) which enrolled more than 4400 CKD patients (not on dialysis) highlight the finding that copeptin independently contributed to prediction of non-cardiovascular death and heart failure hospitalization, even when the NT proBNP and conventional risk factors were taken into consideration [15]. This holds our observed correlation between high copeptin and compromised renal and systemic outcomes.

In 2023, a systematic review by Iglesias et al. showed that copeptin has close ties with microalbuminuria, deteriorating kidney function, and ESRD development in diabetic and even non-diabetic populations [2]. Our ROC cut-offs and correlation signals match pretty closely with these broader, population ones.

High copeptin indicates an enhanced AVP activity supporting renal afferent vasoconstriction, glomerular hyperfiltration, and enhanced tubular sodium and water reabsorption. In turn, it causes albuminuria and chronic renal disorder [16]. This mechanism is highlighted by the strong negative relationships found between copeptin and eGFR in our sample group (*r* = 0.707, *P* < 0.001).

Based on our data, Copeptin should not be seen as an independent diagnostic tool but rather regarded as a supplementary biomarker that could improve risk assessment when analyzed together with well-known glycemic and renal parameters. The identified cutoff values (> 3452 pg/ml poor glycemic control and > 370 pg/ml nephropathy) would give potential values of clinical stratification. Considering the current data on the priority of early glycemic control as the means of preserving renal outcomes in T2DM and preventing the long-term complications (the 2024 glycemic control consensus) [6], including the measure of copeptin would help to increase the detection of the high-risk patients even before the onset of overt albuminuria or deteriorating eGFR is present.

The key strengths of this study are that diabetic patients were thoroughly divided into the groups of controlled and uncontrolled patients with the use of standard HbA1c cutoffs, and that the authors were interested in the connection of both glycemic control and diabetic kidney disease with the help of serum copeptin. Copeptin quantification performed as ELISA creates methodological rigor and laboratory reproducibility.

The limitations of the study, however, are single-centered study and modest sample that can have implications on generalization and statistical determination. Although, the age difference between groups was accounted for by running ACNOVA test, subgroup analysis based on age, sex and menopausal state couldn’t be performed owing to the small sample size. the cross-sectional study design cannot determine whether elevated copeptin precedes or predict the decline of eGFR and renal endpoints. Lastly, lack of longitudinal follow-up will not allow assessing the future renal outcomes based on raised copeptin levels. However, samples will be retained for follow-up measurement plan in future applications. We used 24-hour urinary protein quantification and eGFR to classify kidney involvement; we acknowledge that spot urine albumin-to-creatinine ratio (ACR) is the recommended screening test for DKD (KDIGO guidance) and may detect earlier albuminuria [17]. This omission is a limitation and will be addressed in future studies.

The lack of assessment of inflammatory markers and it’s association state of diabetic control and Copeptin (4&5). Along with the lack of comparing the diagnostic performance of established DKD biomarkers, are possible confounders to the current study results. Notably, NGAL and KIM-1 reflect tubular injury while cystatin C is a filtration marker; copeptin uniquely reflects vasopressin activity and systemic hemodynamic/hormonal stress [18]. Future studies should measure copeptin together with tubular (KIM-1, NGAL) and filtration (cystatin C) markers to assess additive predictive value, using net reclassification and decision-curve analyses.

Subsequent researches should utilize prospective, large cohorts, with numerous sampling locations and to confirm the diagnostic limits, in addition to determining whether high baseline copeptin indicates long-term renal results. Increased hydration or vasopressin receptor antagonism to reduce copeptin may also be worth investigating by interventional trials that could slow DKD in the same way they may do so in early CKD. Furthermore, Elevated copeptin levels were associated with diabetic kidney involvement, representing established but variably severe diabetic kidney disease rather than early subclinical nephropathy, as suggested by recent AI-based models of diabetic nephropathy risk which have reached AUCs of 0.96 or more [19, 20].

## Conclusion

According to our study, the raised serum copeptin highly correlates with bad glycemic control and diabetic kidney disease in type 2 DM. These findings indicate good diagnostic performance of copeptin, its high correlation with major clinical predictive factors, and consistency with the latest evidence, which is evidence supporting the idea that it can be a useful biomarker of early detection and risk stratification. Longitudinal and interventional studies should be conducted in the future in order to determine its causative and treatment significance.

## Data Availability

The datasets used and analyzed during the current study available from the corresponding author on reasonable request.

## References

[1] International Diabetes Federation (IDF). IDF Diabetes Atlas (11th ed.). 2024. https://diabetesatlas.org/.

[2] Iglesias M, González MJ, Álvarez B. Emerging molecular pathways and targets in diabetic kidney disease: from pathophysiology to therapeutic perspectives. Biomolecules. 2023;13(6):845. 10.3390/biom13060845.37238714

[3] Cohen DL, Patel R, Mahadevan G. Copeptin as a surrogate marker of vasopressin: clinical insights into kidney and metabolic disease. Kidney Int Rep. 2024;9(1):210–9. 10.1016/j.ekir.2023.11.002.

[4] Donate-Correa J, Luis-Rodríguez D, Martín-Núñez E, Tagua VG, Hernández-Carballo C, Ferri C, Rodríguez-Rodríguez AE, Mora-Fernández C, Navarro-González JF. Inflammatory targets in diabetic nephropathy. J Clin Med. 2020;9(2):458. 10.3390/jcm9020458. 32046074 10.3390/jcm9020458 PMC7074396

[5] Popovic M, Ebrahimi F, Urwyler SA, Donath MY, Christ-Crain M. The role of IL-1 in the regulation of copeptin in patients with metabolic syndrome. Endocr Connect. 2020;9(7):715–23. 10.1530/EC-20-0197. 32698151 10.1530/EC-20-0197 PMC7424357

[6] El-Soudany S, Farag A, Hegazy M. Evaluation of copeptin as a biomarker in diabetic nephropathy: an Egyptian cohort study. Egypt J Hosp Med. 2023;90(1):2171–7. https://ejhm.journals.ekb.eg/article_181217.html.

[7] Ahmed NM, Zahran MH, Younis A. Role of copeptin as an early predictor of diabetic kidney disease. Zagazig Univ Med J (ZUMJ). 2024;30(2):337–45. https://zumj.journals.ekb.eg/article_337329.html.

[8] Sahu A, Kaur R, Gupta R. Copeptin: A novel biomarker in the spectrum of metabolic syndrome and type 2 diabetes. Front Endocrinol. 2023;14:1124587. 10.3389/fendo.2023.1124587.

[9] Sailer CO, Refardt J, Blum CA, Schnyder I, Molina-Tijeras JA, Fenske W, Christ-Crain M. Validity of different copeptin assays in the differential diagnosis of the polyuria-polydipsia syndrome. Sci Rep. 2021;11(1):10104. 10.1038/s41598-021-89505-9. 33980941 10.1038/s41598-021-89505-9 PMC8114908

[10] Choy KW, Wijeratne N, Chiang C, Don-Wauchope A. Copeptin as a surrogate marker for arginine vasopressin: analytical insights, current utility, and emerging applications. Crit Rev Clin Lab Sci. 2025;62(1):24–44. 10.1080/10408363.2024.2383899. 39086073 10.1080/10408363.2024.2383899

[11] Wang Y, Wang S, Liang S, Zhou X, Guo X, Huang B, Pan H, Zhu H, Chen S. Impact factors of blood copeptin levels in health and disease States. Endocr Practice: Official J Am Coll Endocrinol Am Association Clin Endocrinologists. 2024;30(12):1197–205. 10.1016/j.eprac.2024.09.017. 10.1016/j.eprac.2024.09.017 39357821

[12] Enhörning S, Vanhaecke T, Dolci A, Perrier ET, Melander O. Investigation of possible underlying mechanisms behind water-induced glucose reduction in adults with high copeptin. Sci Rep. 2021;11(1):24481. 10.1038/s41598-021-04224-5. 34966186 10.1038/s41598-021-04224-5 PMC8716535

[13] Henrique LR, Crispim D, Vieceli T, Schaeffer AF, Bellaver P, Leitão CB, Rech TH. Copeptin and stress-induced hyperglycemia in critically ill patients: A prospective study. PLoS ONE. 2021;16(4):e0250035. 10.1371/journal.pone.0250035. 33882083 10.1371/journal.pone.0250035 PMC8059855

[14] Abdelmageed M, Güzelgül F. Copeptin: Up-to-date diagnostic and prognostic role highlight. Anal Biochem. 2023;673:115181. 10.1016/j.ab.2023.115181. 37247750 10.1016/j.ab.2023.115181

[15] German Chronic Kidney Disease (GCKD) Study Investigators. Copeptin as an independent predictor of non-cardiovascular mortality and heart failure in chronic kidney disease patients. Nephrol Dialysis Transplantation. 2023;38(2):456–67. 10.1093/ndt/gfac327.

[16] Kidney Disease: Improving Global Outcomes (KDIGO) Diabetes Work Group. KDIGO 2022 clinical practice guideline for diabetes management in chronic kidney disease. Kidney Int. 2022;102(5S):S1–127. 10.1016/j.kint.2022.06.008. 36272764 10.1016/j.kint.2022.06.008

[17] El Boustany R. Vasopressin and diabetic kidney disease. Ann Nutr Metab. 2018;72(Suppl 2):17–20. 10.1159/000488124. 29925069 10.1159/000488124

[18] Aletras G, Bachlitzanaki M, Stratinaki M, et al. Integrating novel biomarkers into clinical practice: A practical framework for diagnosis and management of cardiorenal syndrome. Life (Basel). 2025;15(10):1540. 10.3390/life15101540. Published 2025 Oct 1. 41157213 10.3390/life15101540 PMC12565375

[19] Lemley KV, Molitch ME. Early intervention in diabetic nephropathy: clinical targets and therapeutic options. Kidney Int Rep. 2024;9(1):99–108. 10.1016/j.ekir.2023.11.003.

[20] Zhang Y, Zhao X, Xu D. Machine learning-based predictive modeling for diabetic nephropathy using metabolic and clinical features. Front Endocrinol. 2024;15:1130156. 10.3389/fendo.2024.1130156.

